# Self-reported immature defense style as a predictor of outcome in short-term and long-term psychotherapy

**DOI:** 10.1002/brb3.190

**Published:** 2014-05-09

**Authors:** Maarit A Laaksonen, Carlos Sirkiä, Paul Knekt, Olavi Lindfors

**Affiliations:** 1 Department of Health, Functional Capacity and Welfare, National Institute for Health and Welfare (THL)Helsinki, Finland; 2 Biomedicum HelsinkiHelsinki, Finland; 3 Social Insurance InstitutionHelsinki, Finland

**Keywords:** Anxiety, depression, outcome, psychotherapy

## Abstract

**Objective:**

Identification of pretreatment patient characteristics predictive of psychotherapy outcome could help to guide treatment choices. This study evaluates patients' initial level of immature defense style as a predictor of the outcome of short-term versus long-term psychotherapy.

**Method:**

In the Helsinki Psychotherapy Study, 326 adult outpatients with mood or anxiety disorder were randomized to individual short-term (psychodynamic or solution-focused) or long-term (psychodynamic) psychotherapy. Their defense style was assessed at baseline using the 88-item Defense Style Questionnaire and classified as low or high around the median value of the respective score. Both specific (Beck Depression Inventory [BDI], Hamilton Depression Rating Scale [HDRS], Symptom Check List Anxiety Scale [SCL-90-Anx], Hamilton Anxiety Rating Scale [HARS]) and global (Symptom Check List Global Severity Index [SCL-90-GSI], Global Assessment of Functioning Scale [GAF]) psychiatric symptoms were measured at baseline and 3–7 times during a 3-year follow-up.

**Results:**

Patients with high use of immature defense style experienced greater symptom reduction in long-term than in short-term psychotherapy by the end of the 3-year follow-up (50% vs. 34%). Patients with low use of immature defense style experienced faster symptom reduction in short-term than in long-term psychotherapy during the first year of follow-up (34% vs. 19%).

**Conclusion:**

Knowledge of patients' initial level of immature defense style may potentially be utilized in tailoring treatments. Further research on defense styles as outcome predictors in psychotherapies of different types is needed.

## Introduction

Both short-term and long-term psychotherapies are common in the treatment of mood and anxiety disorders. Evidence-based knowledge on which treatment is most effective for whom is, however, scarce. Various patient-related variables have been suggested as being essential in the evaluation of patients' suitability for psychotherapies and thus in the prediction of their psychotherapy outcome (Blenkiron 1999; Valbak 2004; Norcross and Wampold 2011). One such variable is the initial level of defense style, that is, availability and integration of individual regulating functions, defense mechanisms, aimed at alleviating anxiety-provoking stressors and maintaining mental balance (American Psychiatric Association 1994; OPD Task Force 2001). Healthy aspects of personality, such as a well-integrated, mature defense style, are considered important predictors of positive outcome of short-term psychotherapies, not aiming to achieve structural changes in personality (Van et al. 2009). On the other hand, patients with a less integrated, immature defense style may need long-term treatments to recover. The use of defense styles as outcome predictors in individual psychotherapies has been little studied to date, only in short-term psychotherapies, and with somewhat contradictory findings (Hersoug et al. 2002; Kronström et al. 2009; Van et al. 2009). No studies comparing the prediction of outcome by defense styles in short-term versus long-term psychotherapies have so far been published. More research on the use of defense styles as predictors of psychotherapy outcomes in general and on potential differential prediction of immature defense style on short-term versus long-term psychotherapy outcome in particular is thus needed.

## Aims of the Study

This study examines the relationship between level of self-reported immature defense style and subsequent changes in psychiatric symptoms in individual short-term and long-term psychotherapy during a 3-year follow-up period. Our aim was to find out whether the level of immature defense style prior to therapy differentiates the outcome of short-term and long-term therapy.

## Material and Methods

This study was part of the Helsinki Psychotherapy Study (HPS). The methods used have been described in detail elsewhere (Knekt and Lindfors 2004; Knekt et al. 2008, 2012) and are summarized here. Patients gave written informed consent. The study protocol was approved by the Helsinki University Central Hospital's ethics council.

### Patients

Outpatients from the Helsinki region were referred to the study by local practitioners from June 1994 to June 2000 (Knekt and Lindfors 2004; Knekt et al. 2008). Eligible patients were 20–45 years of age and had a long-standing (>1 year) disorder causing work dysfunction. Patients were required to meet the DSM-IV criteria (American Psychiatric Association 1994) for anxiety or mood disorders evaluated based on a semi-structured diagnostic interview (Knekt and Lindfors 2004) and Kernberg's criteria (Kernberg 1996) for neurosis to high-level borderline personality organization evaluated based on a psychodynamic assessment interview (Kernberg 1996). Patients with psychotic disorder or severe personality disorder (DSM-IV cluster A personality disorder and/or lower level borderline personality organization), adjustment disorder, substance-related disorder, organic brain disease, or mental retardation were excluded from the study. Patients suffering from such disorders are generally considered to be in need of a longer treatment as they are unlikely able to tolerate considerable anxiety, which is a requirement for a shorter, more anxiety-provoking treatment; therefore their randomization to short-term therapy was not considered fair (American Psychiatric Association 1985; Blenkiron 1999). Patients treated with psychotherapy within the previous 2 years, psychiatric health employees and persons known to the research team members were also excluded.

Altogether 459 patients were considered eligible, of which 133 declined to participate. The remaining 326 patients were randomized according to a central computerized randomization schedule in a 1: 1: 1.3 ratio to individual solution-focused therapy (*N* = 97), short-term psychodynamic psychotherapy (*N* = 101), or long-term psychodynamic therapy (*N* = 128) (Knekt et al. 2008). Of the patients randomized, 33 declined to participate, and 42 of those starting treatment discontinued prematurely.

The patients were followed up for 3 years after randomization. During this 3-year follow-up, the patients were provided, in accordance with the study protocol, with either short-term therapy followed by no treatment, or long-term therapy. Follow-up measurements were carried out at eight occasions (at 0, 3, 7, 9, 12, 18, 24, and 36 months). The mean dropout rates over these eight measurement occasions in the three therapy groups were similar (15% in the solution-focused, 13% in the short-term psychodynamic, and 18% in the long-term psychodynamic therapy group) (Knekt et al. 2008).

### Therapies

Solution-focused therapy is a brief resource-oriented and goal-focused therapeutic approach, helping clients change by constructing solutions (Johnson and Miller 1994; Lambert et al. 1998). The orientation was based on an approach developed by de Shazer et al. (1986), de Shazer (1991). The frequency of sessions was flexible, usually one every second or third week, with a maximum of 12 sessions (90 min each) over no more than 8 months.

Short-term psychodynamic psychotherapy is a brief, focal, transference-based therapeutic approach, helping patients by exploring and working through specific intrapsychic and interpersonal conflicts. The orientation was based on approaches described by Malan (1976) and Sifneos (1978). The therapy was scheduled for 20 treatment sessions (60 min each), one session a week, over 5–6 months.

Long-term psychodynamic psychotherapy is an open-ended, intensive, transference-based therapeutic approach, helping patients by exploring and working through a broad area of intrapsychic and interpersonal conflicts. Therapy includes both expressive and supportive elements, the use of which depends on patient needs. The orientation followed the clinical principles of long-term psychodynamic psychotherapy (Gabbard 2004). The session frequency was 2–3 sessions a week and the duration of therapy up to 3 years.

### Therapists

Altogether 55 therapists participated in the study; six provided solution-focused therapy, 12 short-term psychodynamic psychotherapy, and 41 long-term psychodynamic psychotherapy (Knekt et al. 2008). All the therapists had been trained in the respective therapy form. The mean number of years of experience in the therapy form provided was nine (range 3–15) in solution-focused therapy, nine (range 2–20) in short-term psychodynamic psychotherapy, and 18 (range 6–30) in long-term psychodynamic psychotherapy. Additionally, the therapists providing short-term psychodynamic psychotherapy had a mean of 16 years (range 10–21) experience of long-term psychodynamic psychotherapy. None of the therapists providing solution-focused therapy had received any training in psychodynamic psychotherapy and vice versa. Only solution-focused therapy was manualized, and clinical adherence monitoring was performed. All the solution-focused therapists carried out therapy at a center for solution-focused therapy, in which group supervision was part of the institute procedures. On the contrary, all the psychodynamic therapists were private practitioners who had a variety of different arrangements with regard to (primarily individual) supervision. Both psychodynamic psychotherapies were conducted in accordance with clinical practice, where interventions can be modified to patients' needs within the psychodynamic framework.

### Assessments at baseline

Patient defense style was assessed using a self-report questionnaire at baseline, before randomization of patients into therapies. Other baseline factors potentially confounding the relationship between the baseline defense style and psychotherapy outcome during follow-up were assessed using questionnaires and interviews.

#### Defense style

Psychological defense styles were assessed as a part of personality functions assessment using the Finnish translation (Sammallahti et al. 1994) of the revised 88-item Defense Style Questionnaire (DSQ) by Andrews et al. (1989). Each item consists of an attitudinal statement describing defenses along an ordinal continuum ranging from no agreement (score 1) to total agreement (score 9). A three-factor scale, based on factor analysis (Andrews et al. 1993), has been developed to group the individual defenses into three different types of defense styles: immature, neurotic, and mature (Andrews et al. 1989). This scale has been proven to be an internally reliable instrument, the internal consistency reliabilities (Cronbach's alpha) being 0.89, 0.72, and 0.59 for factors 1, 2, and 3, respectively (Kim and Mueller 1978). Of the 88 items, 72 items are included in the calculation of defense style scores (16 items were control questions); immature, neurotic, and mature defense style scores are based on 46, 16, and 10 items, respectively. The immature defense style covers the specific defenses of acting out, autistic fantasy, denial, devaluation, displacement, dissociation, isolation, passive aggression, projection, rationalization, splitting, and somatization. A score describing the amount of defense style at present is calculated as a mean of the corresponding items, varying thus from 1.0 to 9.0.

A factor analysis was carried out to investigate the internal factor structure of the DSQ in the HPS sample. The three-factor solution resembled the original solution (Kim and Mueller 1978; Andrews et al. 1989), with Cronbach's alphas 0.81, 0.68, and 0.50 for immature, neurotic, and mature defense style factors, respectively. The level of immature defense style in this study was classified as low or high around the median value of the score (3.98); a score under the median represented low use of immature defense style and a score equal to or above the median represented high use of immature defense style.

#### Potential confounding factors

Psychiatric diagnoses at Axes I and II were assessed based on a semi-structured interview (Knekt and Lindfors 2004) according to the DSM-IV diagnostic criteria (American Psychiatric Association 1994). Sociodemographic factors (gender, age, education), psychiatric history (previous depressive states, previous psychotherapy), personality functions, including suitability for psychotherapy (SPS; median kappa coefficient for agreement 0.69 [Laaksonen et al. 2012]), quality of object relations (QORS; Azim et al. 1991), and interpersonal problems (IIP; Horowitz et al. 2000), as well as social functioning, including life orientation (LOT; Scheier and Carver 1985), sense of coherence (SOC; Antonovsky 1993), and social adjustment (SAS-SR; Weissmann and Bothwell 1976) were assessed using questionnaires.

### Assessments at follow-up

The primary outcome measures were specific depressive and anxiety symptoms. The symptoms of depression were assessed with the 21-item self-report Beck Depression Inventory (BDI; Beck et al. 1961) and with the 17-item observer-rated Hamilton Depression Rating Scale (HDRS; Hamilton 1960). The symptoms of anxiety were assessed with the 10-item self-reported Symptom Check List Anxiety Scale (SCL-90-Anx; Derogatis et al. 1973) and with the 14-item observer-rated Hamilton Anxiety Rating Scale (HARS; Hamilton 1959). Secondary outcome measures describing general psychiatric symptoms and global functional capacity were assessed with the Symptom Check List Global Severity Index (SCL-90-GSI; Derogatis et al. 1973) and the Global Assessment of Functioning Scale (GAF; American Psychiatric Association 1994). The self-report measures (BDI, SCL-90-Anx, SCL-90-GSI) were assessed at baseline and 3, 7, 9, 12, 18, 24, and 36 months after the start of the treatment, and the observer-rated measures (HDRS, HARS, GAF) at baseline and 7, 12, and 36 months after the start of the treatment.

### Statistical methods

The statistical analyses were based on linear mixed models (Verbeke and Molenberghs 1997) carried out with SAS software, version 9.1. (SAS Institute Inc 2007). The main analyses were based on the “intention-to-treat” (ITT) design. Complementary “as-treated” (AT) analyses were also performed (Härkänen et al. 2005; Knekt et al. 2008). The primary analyses were based on the assumption of ignorable dropouts (Knekt et al. 2008). In the secondary analyses, missing values were replaced by multiple imputation. The imputation was based on the Markov chain Monte Carlo methods. Model adjusted outcome means and mean differences were calculated for different measurement points (Lee 1981). The delta method was used for the calculation of confidence intervals (Migon and Gamerman 1999). The statistical significance of the model used was tested with the Wald test.

In the ITT analyses, two models were used: a basic model and a complete model. The dependent variable in the analyses was each of the outcome measures (BDI, HDRS, SCL-90-Anx, HARS, SCL-90-GSI, GAF) at a time. The basic ITT model included as independent variables the immature defense style measured at baseline, therapy group, and time (i.e., measurement points), their first- and second-order interactions, a correction term (i.e., the second-order interaction of the difference between theoretical and realized date of measurement, time and immature defense style), and outcome measure at baseline. The complete ITT model further included sociodemographic variables (age, gender, education), DSM-IV diagnoses (Axes I and II, major depressive disorder, and comorbidity of mood and anxiety disorder), psychiatric history data (previous depressive states, previous psychotherapy), personality functions (SPS, QORS, IIP) and social functioning (LOT, SOC, SAS-SR). These were all measured at baseline and satisfied the criteria for confounding, that is, were related with the immature defense style and preceded and were causally related to any of the six outcome measures, without being an intermediate or latent variable (Rothman and Greenland 1998).

To account for the deviations from the study protocol, an AT model was created by adding variables describing noncompliance, that is, waiting time from randomization to initiation of treatment and degree of participation (i.e., withdrawal from or discontinuation of treatment) during follow-up as main effects to the complete ITT model. All three models (ITT basic, ITT complete, AT) were carried out based on both the original data and imputed data. The independent variable of main interest was the interaction term between the immature defense style score, therapy group, and time. As no notable differences in the prediction of the immature defense style on outcome of solution-focused therapy and short-term psychodynamic psychotherapy were found during the 3-year follow-up, these two short-term therapies were combined into one short-term therapy group which was compared to the long-term therapy group. A comparison between the basic and complete ITT models demonstrated that the results were slightly different depending on whether potential confounding factors were used in the statistical model, whereas no major difference between the ITT and AT models were found (data not shown). Imputation caused the confidence intervals to widen, and accordingly resulted in statistical significance of some of the comparisons to disappear (data not shown). The results presented are based on the complete ITT model as based on the original data.

The significance of the immature defense style in predicting the outcome of short-term versus long-term therapy during the 3-year follow-up was evaluated by testing the statistical significance of the interaction term between the immature defense style and the therapy group throughout the follow-up. The Wald test was used.

We assessed statistical significance of the change in outcome from baseline to the different measurement points for each therapy group (short-term and long-term) and category (low and high) of immature defense style. Therapy was considered beneficial for the patients who experienced and maintained a statistically significant reduction in symptoms in comparison with the baseline during the 3-year follow-up.

We measured the statistical significance of the model-adjusted difference in the outcome between the therapy groups in the immature defense style categories at the different measurement points. We considered short-term therapy to be equally or more beneficial than long-term therapy when there were no statistically significant differences between the therapy groups or greater benefit from short-term therapy, whereas long-term therapy was considered to be more beneficial when comparisons favored long-term over short-term therapy.

## Results

The study sample consisted of 326 patients ranging in age from 20 to 46 years (mean 32 years) (Table 1). Approximately 25% of the patients were men, about 50% lived alone, and 25% had an academic education. The majority (85%) of the patients suffered from mood disorder, 44% from anxiety disorder, and 18% from personality disorder. No notable differences between short-term and long-term therapy groups were found with respect to baseline sociodemographic or clinical characteristics, with the exception of significantly higher prevalence of anxiety disorder and personality disorder in the short-term therapy group.

**Table 1:** Baseline characteristics of the 326 patients intended to treat by treatment group

	Treatment^1^	
	
Characteristic	Short (*N* = 198)	Long (*N* = 128)
Sociodemographic variables		
Men (%)	25.8	21.1
Age (years)^2^	32.8 (7.1)	31.6 (6.6)
University degree (%)	24.2	28.1
Living alone (%)	52.5	49.2
Employed (%)^3^	84.2	75.4
Psychiatric diagnoses^4^		
Mood disorder (%)	82.3	88.3
Anxiety disorder (%)	48.0	36.7
Comorbid mood and anxiety disorder (%)	30.3	25.0
Personality disorder (%)	21.7	12.5
Psychiatric symptoms		
Beck Depression Inventory (BDI)^2^	18.0 (7.6)	18.8 (8.3)
Hamilton Depression Rating Scale (HDRS)^2^	15.6 (4.7)	15.8 (4.9)
Symptom Check List, Anxiety scale (SCL-90-Anx)^2^	1.3 (0.7)	1.2 (0.7)
Hamilton Anxiety Rating Scale (HARS)^2^	15.0 (5.3)	14.8 (5.2)
Symptom Check List, Global Severity Index (SCL-90-GSI)^2^	1.3 (0.5)	1.3 (0.6)
Global Assessment of Functioning Scale (GAF)^2^	55.1 (7.1)	55.5 (8.1)
Psychiatric background		
Recurrent episodes of major depressive disorder (%)	64.1	69.1
Duration of primary disorder over 5 years (%)	34.3	29.7
Previous psychotherapy (%)	19.4	19.0
Psychological defenses (DSQ)		
Immature defense style^2^	3.93 (0.76)	3.93 (0.69)
Neurotic defense style^2^	4.21 (0.96)	4.21 (0.94)
Mature defense style^2^	5.25 (0.92)	5.12 (1.01))

1 Short, Short-term psychodynamic psychotherapy and solution-focused therapy combined; Long, Long-term psychodynamic psychotherapy.

2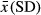.

3 Full-time/part-time work or full-time student/student at work.

4 Mood disorder: mood disorder only or comorbid mood and anxiety disorder. Anxiety disorder: anxiety disorder only or comorbid mood and anxiety disorder. Personality disorder: main diagnoses on Axis II.

During the 3-year follow-up, a statistically significant symptom reduction was found in all six outcome measures (BDI, HDRS, SCL-90-Anx, HARS, SCL-90-GSI, GAF) in both short-term and long-term therapy groups (Table 2). There was no statistically significant interaction between immature defense style and therapy group in the 3-year follow-up. Numerous consistent statistically significant differences in symptom development between short-term and long-term therapies at different points of follow-up were, however, found in relation to the level of immature defense style. During the first year of the follow-up, a statistically larger symptom reduction in the short-term therapy group than in the long-term therapy group (18–45% vs. 9–31%, respectively) was found among patients with low immature defense style according to all six outcome measures. At the end of the 3-year follow-up, on the other hand, a statistically larger symptom reduction in the long-term therapy group than in the short-term therapy group (26–64% vs. 21–45%, respectively) was found among patients with high immature defense style according to five outcome measures.

**Table 2:** Mean values and mean value differences (95% confidence intervals) between short-term and long-term therapy at baseline and at 7, 12, and 36 month follow-up according to the low and high values of the immature defense style score.^1961^

		Low^2^				High^2^				
				
Outcome measure	Therapy	0	7	12	36	0	7	12	36	*P*-value^3^
BDI	Short	18.0	10.7	10.1	10.0	18.0	9.91	9.55	10.1	0.50
	Long	18.2	14.7	12.7	7.58	19.2	14.1	12.8	6.47	
	S-L^4^		−3.96* (−6.57, −1.35)	−2.60 (−5.36, 0.15)	2.43 (−0.49, 5.35)		−4.23* (−6.79, −1.67)	−3.26* (−5.99, −0.52)	3.63* (0.62, 6.65)	
HDRS	Short	15.3	10.7	10.6	10.7	15.9	11.1	11.1	10.7	0.26
	Long	15.5	13.3	13.8	10.3	16.2	12.4	11.7	7.87	
	S-L^4^		−2.57* (−4.40, −0.75)	−3.23* (−5.16, −1.29)	0.47 (−1.54, 2.47)		−1.28 (−3.12, 0.57)	−0.68 (−2.66, 1.30)	2.87* (0.77, 4.97)	
SCL-90-Anx	Short	1.20	0.81	0.81	0.75	1.27	0.94	0.85	0.83	0.18
	Long	1.08	1.10	0.96	0.74	1.38	1.03	0.98	0.53	
	S-L^4^		−0.29* (−0.51, −0.07)	−0.15 (−0.36, 0.05)	0.01 (−0.12, 0.23)		−0.10 (−0.31, 0.12)	−0.13 (−0.33, 0.07)	0.30* (0.09, 0.52)	
HARS	Short	14.4	10.0	9.90	9.71	15.4	10.7	10.5	9.92	0.11
	Long	14.5	13.0	12.5	9.08	15.5	10.9	10.5	7.56	
	S-L^4^		−2.97* (−4.68, −1.27)	−2.55* (−4.33, −0.77)	0.63 (−1.20, 2.46)		−0.21 (−1.93, 1.52)	0.05 (−1.77, 1.87)	2.35* (0.44, 4.26)	
SCL-90-GSI	Short	1.18	0.88	0.82	0.84	1.34	0.92	0.82	0.83	0.54
	Long	1.20	1.14	1.04	0.81	1.38	1.04	0.95	0.60	
	S-L^4^		−0.26* (−0.43, −0.09)	−0.21* (−0.37, −0.05)	0.03 (−0.15, 0.20)		0.12 (−0.29, 0.05)	−0.13 (−0.29, 0.03)	0.23* (0.04, 0.41)	
GAF	Short	54.0	65.3	65.5	67.5	56.3	65.1	66.0	66.8	0.79
	Long	54.3	61.3	60.4	68.4	55.8	63.3	62.9	69.9	
	S-L^4^		4.03* (0.59, 7.46)	5.19* (1.56, 8.83)	−0.88 (−4.28, 3.07)		1.77 (−1.70, 5.23)	3.09 (−0.62, 6.80)	−3.05 (−7.18, 1.09)	

BDI, Beck Depression Inventory; HDRS, Hamilton Depression Rating Scale; SCL-90-Anx, Symptom Check List, Anxiety scale; HARS, Hamilton Anxiety Rating Scale; SCL-90-GSI, Symptom Check List, Global Severity Index; GAF, Global Assessment of Functioning Scale. Underlined symptoms have changed statistically significantly in comparison with baseline symptom level.

* Bold values (*P* < 0.05) are for comparison between short-term and long-term therapy.

1 Complete “intention-to-treat” (ITT) model: outcome measure = interaction of immature defense style score (low, high), therapy group (short, long) and time (in months) adjusted for time, the immature defense style according to the therapy group, the difference between planned and realized date of measurement, second-order interaction of the difference between planned and realized date of measurement, time and immature defense style according to the therapy group, age, gender, education, DSM-IV Axis I diagnosis, DSM-IV Axis II diagnosis, DSM-IV major depressive disorder diagnosis, comorbidity of mood and anxiety disorder, previous depressive states, previous psychotherapy, suitability for psychotherapy, quality of object relations, interpersonal problems, life orientation, sense of coherence, social adjustment, and the baseline level of the outcome measure.

2 The median score of the immature defense style in the Helsinki Psychotherapy Study sample was 3.98. Low immature defense style was categorized as values below the median (2.00–3.97) and high immature defense style as values equal to or above the median (3.98–6.04).

3 *P*-value for interaction between the immature defense style, the therapy group, and time throughout the follow-up.

4 Mean value difference of outcome measure between short-term and long-term psychotherapy.

Imputation, carried out to study the reliability of the ignorable drop-out assumption, attenuated the results to some extent but the benefit of short-term therapy during the first year of follow-up still remained statistically significant for five of six outcome measures (excluding GAF) and the benefit of long-term therapy during the last year of follow-up for three of five outcome measures (excluding HDRS and HARS) (data not shown). Thus, according to our criteria, for patients with low use of immature defense style short-term therapy seemed to be more beneficial, whereas for patients with high use of immature defense style long-term therapy seemed more beneficial.

## Discussion

It has been suggested that healthy aspects of personality are necessary precursors for positive outcome in short-term psychotherapies which, due to the limited amount of time, in contrast to long-term psychotherapies, do not usually aim at achieving structural changes in personality (Van et al. 2009). It follows that persons with poorly functioning personality are likely to need long-term psychotherapies to recover, as long-term therapies foster capacity for growth and aim to increase self-awareness and to improve interpersonal skills through changes in personality (American Psychiatric Association 1985). A person's characteristic defenses represent one key aspect of their personality organization, manifested in the person's interpersonal behavior and personal experiences. Accordingly, the presence of immature defense style highlights the likelihood that the person is characterized also of having contradictory personality traits, low level of object relations, and an unstable sense of self and others (Kernberg 1996). An immature defense style, defined as a type of maladaptive behavior pattern in which the very occurrence of the perceived psychic threat (affect, idea, or aspect of a relationship) is denied, split-off from consciousness, or in other ways significantly distorted, thus reflects a poorly functioning, that is, more primitive, personality organization. The few previous studies on the analysis of defense styles and overall defensive functioning as predictors of treatment outcome have focused on short-term psychodynamic psychotherapy alone or in comparison to medication and have produced contradictory findings (Hersoug et al. 2002; Kronström et al. 2009; Van et al. 2009). According to Van et al. (2009), self-reported mature, but not neurotic or immature, defense style was predictive of positive symptomatic outcome in short-term psychodynamic psychotherapy. However, according to Kronström et al. (2009) and Hersoug et al. (2002), respectively, mature defense style and overall level of defenses were not predictive of psychotherapy outcome. The role of immature defense style in the prediction of long-term psychotherapy outcome or potential differential prediction of short-term versus long-term psychotherapy outcome has not been previously studied.

In this study, the use of self-reported baseline immature defense style as a predictor of outcomes of short-term and long-term psychotherapies was examined for the first time. No differences in relationship between self-reported immature defense style and outcome in short-term psychodynamic psychotherapy and solution-focused therapy were found. This is in line with our previous findings on other psychotherapy suitability variables (Laaksonen et al. 2013a). The prediction, however, appeared to vary depending on the length of the treatment and follow-up. On average, patients with low use of immature defense style recovered faster in short-term than in long-term psychotherapy, whereas patients with high use of immature defense style recovered better in long-term than in short-term psychotherapy by the end of the 3-year follow-up. Our findings thus support the hypotheses presented in the literature and are also in line with our previous findings (Laaksonen et al. 2013b).

This study has several strengths. First, the relatively large sample size enabled more reliable detection of possible differences. Second, the long follow-up time and frequent outcome measurements allowed a comprehensive description and comparison of the symptom development in the treatment groups. Third, use of various well-validated, both observer-rated and self-reported, outcome measures permitted evaluation of the generalizability of the phenomenon studied. The main results were found according to all these measures, indicating that the phenomenon was independent of different assessment methods. Fourth, a widely used self-report measure of defense styles, DSQ, was used. The measure has been concluded to be the best self-reported defense style assessment method based on available information on the validity and reliability of different methods (Soultanian et al. 2005). Further use also of observer-rated methods would, however, likely give a more comprehensive picture of the predictive role of defense styles, especially as defenses are automatic psychological processes that individuals often are unaware of, and therefore the self-report methods only reflect their conscious derivates and might be sensitive to the influence of the actual psychopathology (Andrews et al. 1989; Van et al. 2009). Fifth, a factor analysis was carried out to compare the internal factor structure of the DSQ in the HPS sample to the original factor solution by Andrews et al. (1989), and the DSQ was concluded to be a valid and reliable measure of immature defense style in the HPS. Sixth, comprehensive criteria, based on statistical significance, for the evaluation of different aspects of prediction of the influence of defense styles on psychotherapy outcome were applied (Laaksonen et al. 2013a,b).

This study has, however, also several limitations. The general limitations related to the design of the HPS (i.e., lack of manuals and blindness of raters making follow-up assessments) are discussed in more detail elsewhere (Knekt et al. 2008, 2012), and only the limitations specific to this study are addressed here. First, although potential confounding factors were comprehensively studied and adjusted by modeling, the possibility of residual confounding cannot be fully excluded. Second, the compliance of study treatment or auxiliary treatment may potentially cause bias (Knekt et al. 2011). Results from AT analysis, adjusted for withdrawal or discontinuation and for time-dependent variables on auxiliary treatment, did not, however, notably differ from the results from ITT analysis. Third, although analyses were carried out based on both the original and imputed data, possible nonignorable dropouts may still bias the results (Härkänen et al. 2005). Some differences between the analyses based on the original and imputed data were found which also emphasizes need for further research. Fourth, a follow-up longer than 3 years is needed to be able to verify the stability of the findings in long-term therapy.

## Concluding Remark

This study suggests that patients with low use of immature defense style may be more suitable for short-term psychotherapy, while patients with high use of immature defense style may benefit more from long-term psychotherapy. More research is, however, needed to confirm these findings and to demonstrate their usefulness in practice.

## Significant Outcomes

1. Symptoms of patients with high use of immature defense style prior to therapy reduced significantly more in long-term than in short-term psychotherapy.
2. Symptoms of patients with low use of immature defense style prior to therapy reduced significantly faster in short-term than in long-term psychotherapy.
3. Level of patient's initial immature defense style appeared to differentiate the suitability of short-term and long-term psychotherapy and may be utilized to guide treatment choices to better match patients to therapies.

## Limitations

1. A widely used and well-validated self-report defense style measure, DSQ, was used for the assessment of immature defense style, but use of observer-rated methods would provide a more comprehensive view of these phenomena.
2. The possible changes in the use of immature defense style during the therapies were not analyzed and thus more research is needed to find out whether the greater benefit of long-term therapy among those with high initial use of immature defense style was due to its gradually lesser use.
3. To be able to tailor treatments more accurately, the research on psychotherapy suitability should be expanded to include a greater variety of patient, therapist and alliance factors and their combinations as well as in the context of psychotherapies of different orientation and length.
